# Thymic Lymphoepithelial Carcinoma: A Rare Aggressive Mediastinal Mass

**DOI:** 10.7759/cureus.64337

**Published:** 2024-07-11

**Authors:** Sumana Kedilaya, RB Revanth, Swetha Balije

**Affiliations:** 1 Onco-Imaging, HealthCare Global Enterprises Ltd., Bangalore, IND

**Keywords:** lymphoepithelioma-like thymic carcinoma, masaoka staging, anterior mediastinal mass, squamous cell carcinoma, thymic carcinoma

## Abstract

Primary thymic lymphoepithelial carcinoma is a rare neoplasm of the mediastinum. The recognition of clinical, radiologic, and pathologic features can help in making an accurate diagnosis of this aggressive tumor at the earliest.Here, we present the radiologic and pathologic findings of an anterior mediastinal mass in a 44-year-old man, which turned out to be a poorly differentiated squamous cell carcinoma of thymic origin, also known as lymphoepithelioma-like thymic carcinoma.

## Introduction

Primary thymic lymphoepithelial carcinoma is a rare neoplasm of the mediastinum and belongs to the malignant spectrum of thymic tumors [1]. Patients usually present with vague chest symptoms or tend to be asymptomatic. They are aggressive invasive neoplasms, with a poor prognosis compared to other mediastinal tumors [2]. In contrast to the more common thymoma, thymic carcinoma tends to have nonspecific diagnostic characteristics. Hence, imaging with a CT scan or PET scan along with correlation with histopathologic features play a crucial role in identifying the lesion at the earliest.

## Case presentation

A 44-year-old male patient came to the hospital with a persistent cough and chest discomfort for the past month. He did not complain of fever, weight loss, or dyspnea. He had no history of smoking.

**Figure 1:**
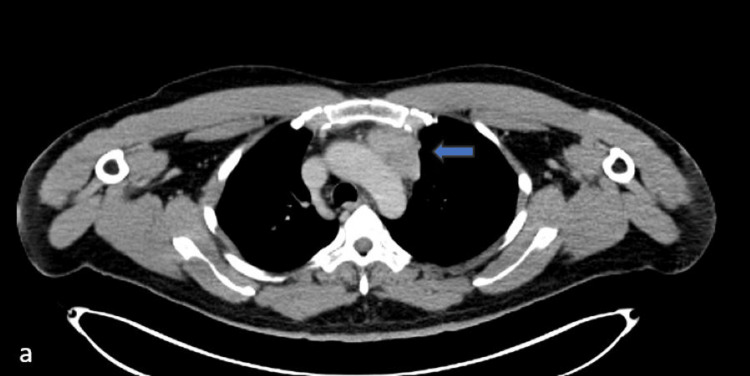
Axial contrast-enhanced computed tomography (CECT) image of the chest at the level of the aortic arch

**Figure 2:**
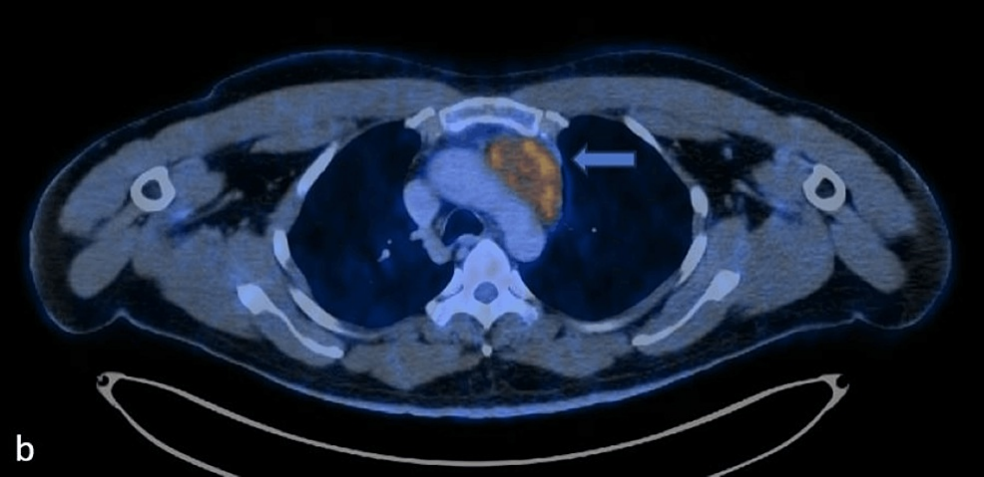
Axial-fused positron emission tomography–computed tomography (PET CT) image of the chest at the level of aortic arch A and B show the metabolically active heterogeneously enhancing mass in the anterior mediastinum abutting the aortic arch.

**Figure 3:**
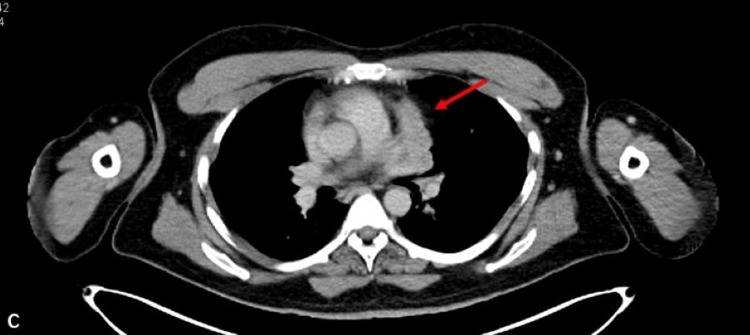
Axial contrast-enhanced computed tomography (CECT) of the chest at the level of the pulmonary artery

**Figure 4:**
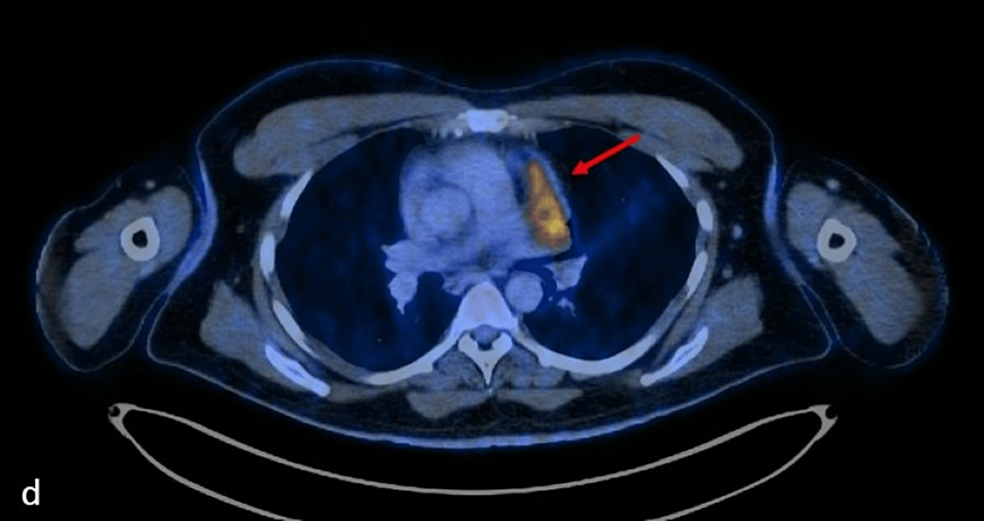
Axial-fused positron emission tomography–computed tomography (PET CT) image of the chest at the level of the pulmonary artery (c,d) show the lesion abutting the main pulmonary artery and left pulmonary artery with loss of fat planes

**Figure 5:**
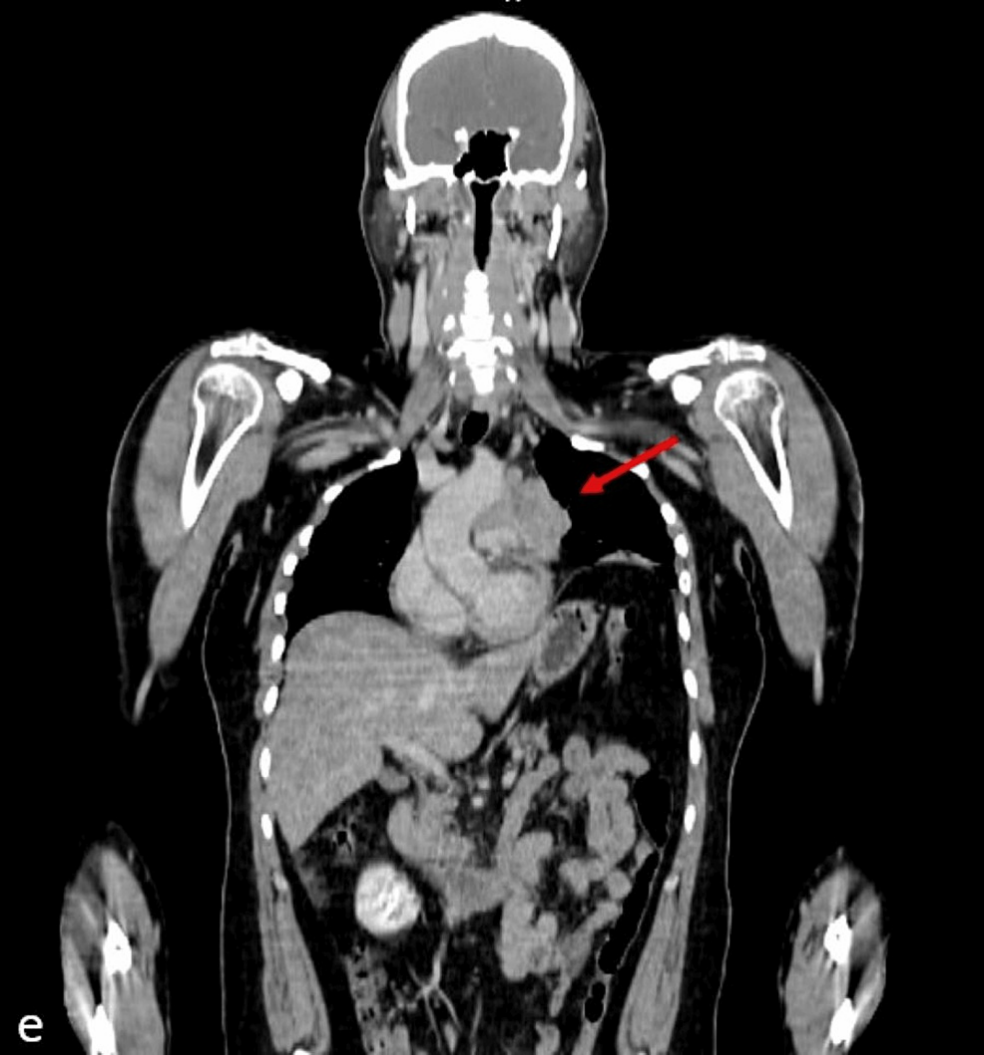
Coronal contrast-enhanced computed tomography (CECT) image of the chest and abdomen

**Figure 6:**
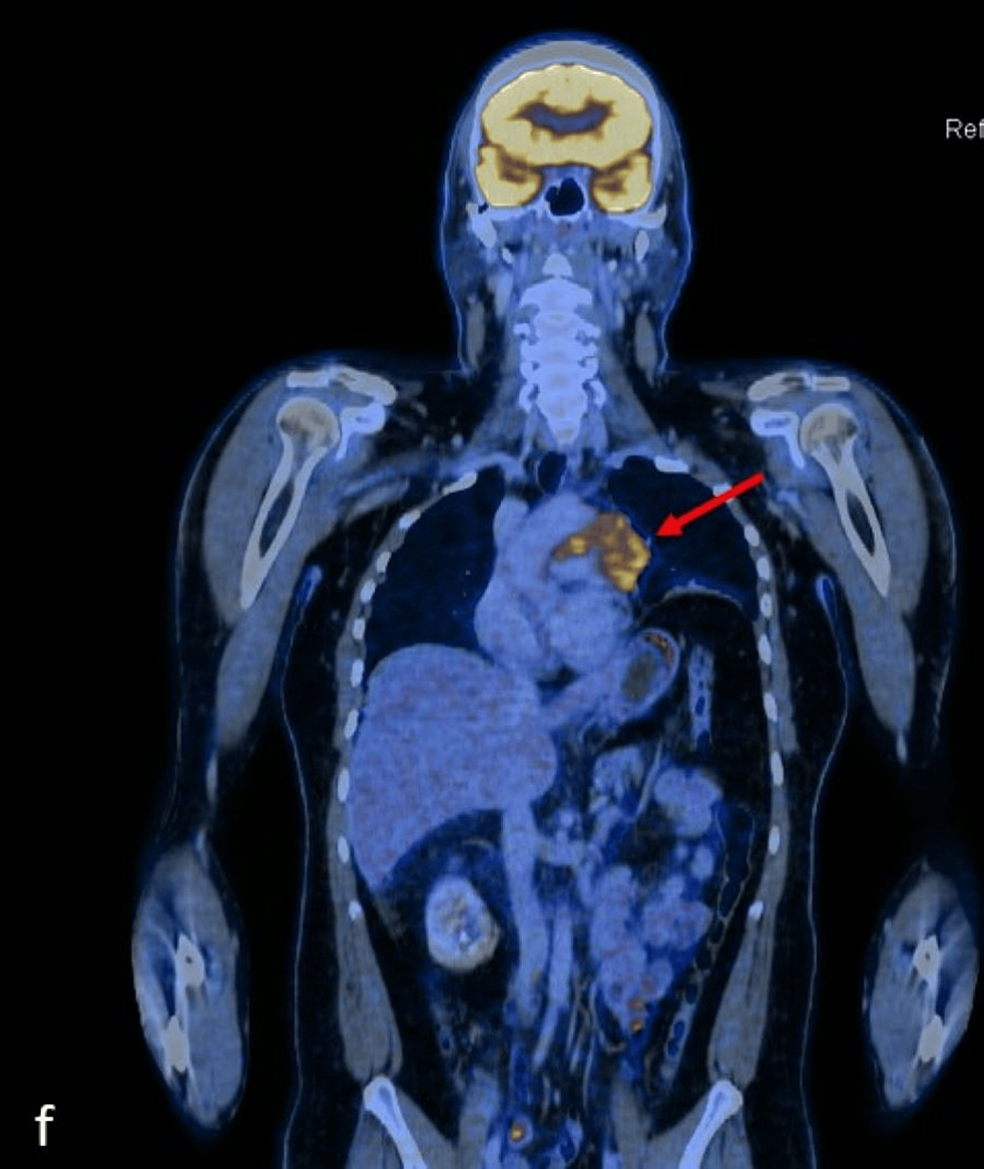
Coronal-fused positron emission tomography–computed tomography (PET CT) image of the chest and abdomen E and F show the elevated left dome of the diaphragm and thin subsegmental atelectasis of the lower lobe of the left lung, secondary to phrenic nerve invasion by the mediastinal mass.

He was initially evaluated outside with a chest X-ray, which revealed mediastinal widening. Following this, a mediastinal mass was suspected, and he was advised to undergo a fludeoxyglucose-18 (FDG) PET CT scan at our department to help characterize the lesion and stage. The PET CT showed a heterogeneously enhancing metabolically active mass lesion in the anterior mediastinum measuring 7.4 x 5.6 cm in transaxial dimensions and 5.7 cm craniocaudally with a standardized uptake value (SUV) of 5.7, as can be seen in Figure 1 and Figure 2. The mass was seen abutting the main pulmonary artery, left pulmonary artery, and arch of the aorta (Figure 3 and Figure 4). No evidence of vascular narrowing was found. Few necrotic areas were seen within the mass. No fat density or calcifications were present within the lesion. There was also an elevation of the left hemidiaphragm with subsegmental atelectasis of the lower lobe of the left lung, indicating left phrenic nerve palsy (Figure 5 and Figure 6). There were no discrete enlarged lymph nodes. There were no lesions in other organs. With these findings, we gave a differential diagnosis of thymic neoplasm versus lymphoma.

To rule out the possibility of germ cell tumors, baseline serum tumor markers were also performed, which were found to be within the normal range as follows: alpha fetoprotein (AFP): 3.47, beta HCG (human chorionic gonadotrophin): <2.39, and lactate dehydrogenase (LDH): 236

**Figure 7:**
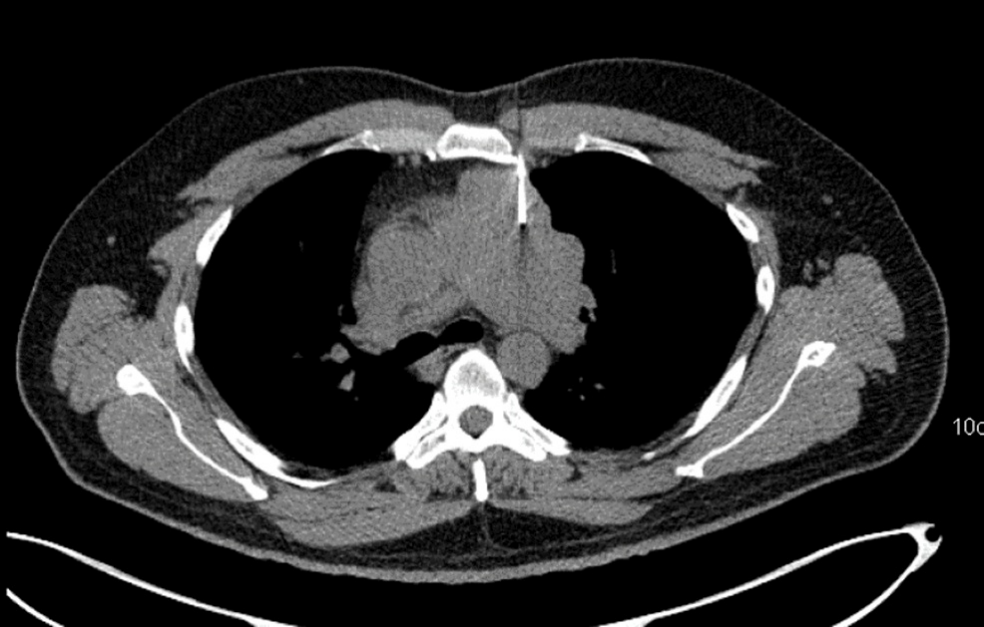
CT-guided biopsy

Following this, a CT-guided biopsy of the mediastinal mass was performed in our department (Figure 7).

**Figure 8:**
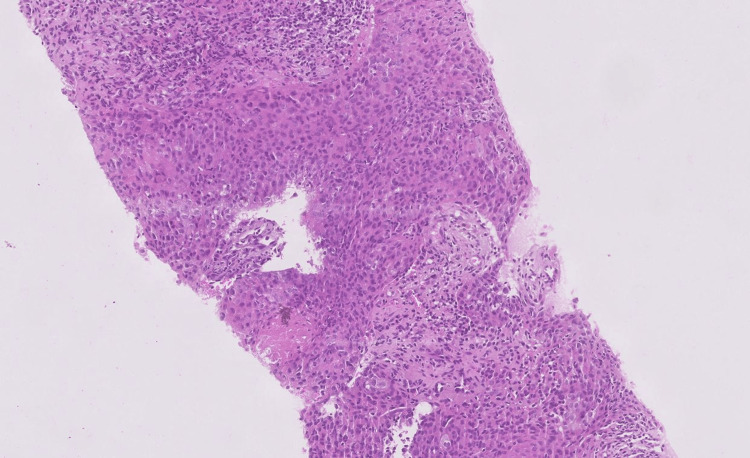
High-power field of the tissue core The image showed that the tumor tissue is composed of neoplastic cells arranged in sheets. The cells have a hyperchromatic nucleus and moderate eosinophilic cytoplasm.

Histopathological sections revealed that the cores of the tumor tissue were composed of neoplastic cells arranged in sheets. The neoplastic cells were predominantly epithelial cells consisting of a hyperchromatic nucleus and moderate eosinophilic cytoplasm. Stroma consists predominantly of mature lymphocytes and shows moderate inflammation (hot tumor) (Figure 8).

**Figure 9:**
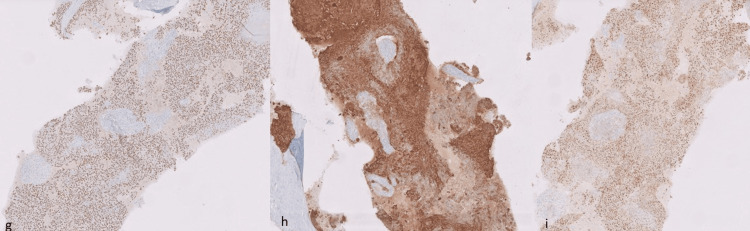
Immunohistochemistry slides are positive for (g) p40, (h) panCK, and (i) pax8.

In addition, immunohistochemistry was done. The tumor cells were positive for p40, PAX8, and panCK (Figure 9) and negative for CK7, CK20, TTF1, c-kit, CD5, synaptophysin, chromogranin, and p16. Hence, the final diagnosis was poorly differentiated squamous cell carcinoma of thymic origin, also known as lymphoepithelioma-like thymic carcinoma.

Since the tumor was seen infiltrating the phrenic nerve, abutting the mediastinal vasculature with loss of fat planes, and no radiologic evidence of nodal or distant metastases, the stage was T4N0M0. In view of the advanced stage, the patient has been started on a chemotherapy regimen of carboplatin and paclitaxel. Thus far, the patient has completed two cycles of chemotherapy, and there has been an improvement in his symptoms.

## Discussion

Lymphoepithelioma-like thymic carcinomas are uncommon primary tumors of the thymus [3].

Thymic neoplasms are broadly categorized into thymomas and thymic carcinomas. Contrary to thymomas, thymic carcinomas are clearly malignant epithelial lesions with pathological features of cellular atypia and invasive margins. Thymic carcinomas have an incidence of 0.07 to 0.38/100,000 per year [4]. Thymomas, on the other hand, include benign lesions and low-grade malignant tumors. The morphologic spectrum of thymic carcinoma is heterogeneous and varied, with the most common being squamous cell carcinoma, accounting for 80% of thymic carcinomas, followed by lymphoepithelial-like variant, which accounts for 6% of thymic carcinomas [5].

Thymic carcinomas typically occur in patients with an average age of 54 to 65 years [4]. Patients tend to be asymptomatic or present with symptoms related to compression or invasion of adjacent mediastinal structures. The common symptoms include vague chest pain, breathing difficulty, chest discomfort, and persistent cough. 

Thymic carcinomas can cause superior vena cava (SVC) syndrome due to mechanical obstruction. In cases where there is gradual obstruction, collateral pathways open up. In an acute setting, diffuse swelling of the face, neck, upper limbs, and chest occurs along with symptoms, such as dyspnea and cough.

Paraneoplastic syndromes can rarely occur in thymic carcinoma cases. The most common is myasthenia gravis. Others include pure red aplasia, hypogammaglobulinemia, and even systemic lupus erythematosus [6].

Radiologically, thymic carcinomas are seen as anterior mediastinal masses. On CT, they are seen as poorly marginated ill-defined hypodense anterior mediastinal masses. Areas of necrosis, cyst formation, and hemorrhage are often present, contributing to heterogeneous enhancement. Calcifications can also be seen. Thymic lymphoepithelial carcinomas have a high invasive nature, due to which contiguous extension along adjacent mediastinal structures is often seen [5]. They can spread to other intrathoracic sites, such as pleural and pericardial lining, and also metastasize to distant organs. Lymph node metastases can be seen in 26% of the cases [7]. CT findings can help categorize and predict the gross histology of thymic neoplasms according to the WHO classification scheme for thymic epithelial tumors [8]. Type A tumors are usually seen as well-defined lobulated soft tissue attenuation tumors on CT, and histologically they are thymomas. Type B tumors more often demonstrate calcifications on CT. Thymic carcinomas fall under type C tumors and are characterized by larger size and invasion of mediastinal fat and other structures. However, it is not possible to differentiate the various histological subtypes of thymic carcinoma since they tend to have similar imaging features.

The differentials for thymic carcinoma include other more common anterior mediastinal lesions, such as thymomas, teratomas, lymphomas, and lung cancer with contiguous mediastinal involvement. Thymomas are usually well-defined lobulated masses. Teratomas tend to have internal areas of fat density along with calcifications. The presence of fat excludes the diagnosis of thymic carcinoma. The presence of other enlarged nodal groups should suggest the possibility of lymphoma.

On PET CT, thymic carcinomas are metabolically active and often have SUV values greater than 5 [9].

The Masaoka staging system is a surgical staging system, used for thymomas and thymic carcinomas [10]. Stage 1 refers to tumors with intact capsules, stage 2 refers to tumors that have capsular invasion into adjacent mediastinal fat, stage 3 refers to macroscopic invasion into adjacent organs, stage 4a refers to dissemination into the thoracic cavity in the form of pleural or pericardial implants, and stage 4b refers to distant metastases.

Since thymic carcinomas tend to be extremely invasive, surgical resectability is less likely in most cases. Surgery may be attempted in cases that seem feasible. Treatment options such as radiation and chemotherapy are generally used due to the aggressive nature of this lesion. The commonly used chemotherapy regimens are platinum-based agents such as cisplatin or carboplatin along with other agents like etoposide or paclitaxel [4]. Five-year survival ranges from 30% to 50% [11]. Lifelong surveillance is recommended due to the high chance of recurrence.

## Conclusions

Thymic lymphoepithelial carcinomas are invasive and aggressive tumors, not often seen in clinical practice. Hence, the identification of the imaging and pathologic features of these tumors is essential for diagnosis. Since their imaging and histopathological features tend to be non-specific, diagnosis is often made based on correlation between the two. Imaging features such as heterogeneous attenuation of anterior mediastinal mass with invasion of adjacent mediastinal structures should raise the suspicion of thymic carcinoma. The treatment of choice includes a multidisciplinary approach with surgery, chemotherapy, and radiation.
